# Highly Efficient SnIn_4_S_8_@ZnO Z-Scheme Heterojunction Photocatalyst for Methylene Blue Photodegradation

**DOI:** 10.3390/ma16196380

**Published:** 2023-09-24

**Authors:** Qiang Luo, Changlin Sun, Juan Zhao, Qizhou Cai, Shanshan Yao

**Affiliations:** 1 School of Mechanical Engineering, Wuhan Polytechnic University, Wuhan 430048, China; luoqiangncwh30@163.com (Q.L.); 15855550526@163.com (C.S.); 2 School of Mathematics & Computer Science, Wuhan Polytechnic University, Wuhan 430048, China; 3 State Key Laboratory of Materials Processing and Die & Mould Technology, Huazhong University of Science and Technology, Wuhan 430074, China; caiqizhou@hust.edu.cn; 4 Institute for Advanced Materials, Jiangsu University, Zhenjiang 212013, China; yaosshan@hotmail.com

**Keywords:** SnIn_4_S_8_@ZnO, Z-scheme heterojunction, hydrothermal synthesis, MB photodegradation

## Abstract

Building heterojunctions is a promising strategy for the achievement of highly efficient photocatalysis. Herein, a novel SnIn_4_S_8_@ZnO Z-scheme heterostructure with a tight contact interface was successfully constructed using a convenient two-step hydrothermal approach. The phase composition, morphology, specific surface area, as well as photophysical characteristics of SnIn_4_S_8_@ZnO were investigated through a series of characterization methods, respectively. Methylene blue (MB) was chosen as the target contaminant for photocatalytic degradation. In addition, the degradation process was fitted with pseudo-first-order kinetics. The as-prepared SnIn_4_S_8_@ZnO heterojunctions displayed excellent photocatalytic activities toward MB degradation. The optimized sample (ZS800), in which the molar ratio of ZnO to SnIn_4_S_8_ was 800, displayed the highest photodegradation efficiency toward MB (91%) after 20 min. Furthermore, the apparent rate constant of MB photodegradation using ZS800 (0.121 min^−1^) was 2.2 times that using ZnO (0.054 min^−1^). The improvement in photocatalytic activity could be ascribed to the efficient spatial separation of photoinduced charge carriers through a Z-scheme heterojunction with an intimate contact interface. The results in this paper bring a novel insight into constructing excellent ZnO-based photocatalytic systems for wastewater purification.

## 1. Introduction

With the rapid development of human society, the problem of pollution is becoming more and more serious, and people have adopted many methods to deal with this problem [1,2,3]. Photocatalytic technology, as a new low-consumption, renewable and non-secondary solution to pollution problems, has received extensive attention from researchers [4,5,6,7,8]. Through the continuous efforts of researchers, photocatalytic materials have been greatly expanded, including oxides and sulfides such as ZnO, CdS, NiCo_2_O_4_ and CuInS_2_ [9,10,11,12], perovskite such as CsPbCl_3_ [13], graphite-like phase g-C_3_N_4_ [14], mono-elemental red phosphorus [15], and so on. Among numerous photocatalytic materials, ZnO is a promising photocatalyst because it possesses many excellent characteristics, such as high electron mobility, low cost, nontoxicity and easy preparation [16,17]. However, it is very easy for the photoinduced electrons and holes of ZnO to recombine, which brings a low quantum yield, thus limiting its photocatalytic activity. For the purpose of inhibiting the recombination between the photoinduced electrons and holes in ZnO, the following methods have been investigated by researchers: (i) Doping metal or non-metallic ions into ZnO can change the electron-hole concentration, thereby inhibiting the recombination between the photoinduced electrons and holes [18,19]. However, it is usually difficult for doping elements to be doped into the lattice of ZnO, and improper doping actually inhibits its activity. (ii) The deposition of noble metals, such as Pt, Ag and Au, on the surface of ZnO is a typical surface modification strategy for the improvement of optical quantum efficiency [20,21]. However, the high cost of these precious metals is not conducive to the promotion and practical application of this technology. (iii) The technique of coupling ZnO with other semiconductors that possess proper energy bands produces heterogeneous structures. The photoinduced charge carriers are separated through directional transfer in the presence of potential difference or the internal electric field generated by the heterojunction, which results in the significant improvement of the photocatalytic efficiency of ZnO [22,23]. Therefore, the construction of heterojunction is a very promising technology for facilitating the progress of ZnO photocatalytic research [24,25,26].

Snln_4_S_8_, a typical polymetallic sulfide, has shown great latent capacity in the fields of photocatalysis due to its unique structure. The SnIn_4_S_8_ material has a primary structure of nanoparticles and a secondary structure of a micron-sized monodisperse sphere constructed from a primary structure. This novel structure has two huge advantages for application in the field of photocatalysis: (i) the primary structure provides a large specific surface area to adsorb more dye molecules; (ii) the secondary and porous structure can act as a light scattering center to improve the utilization of light. So far, Snln_4_S_8_ has been successfully prepared and applied to the photocatalytic degradation of organic pollutants [27]. Most notably, benefiting from the appropriate structure of the Snln_4_S_8_ energy band, the photocatalytic activities of some materials can be enhanced by building a heterojunction with Snln_4_S_8_ [28,29,30]. However, investigations on the application of the Snln_4_S_8_/ZnO heterojunction in the field of the photocatalytic degradation of effluent containing organic contaminant have not yet been reported.

As a common organic dye, methylene blue (MB) is one of the main components of wastewater from the printing and dyeing industry. Wastewater-containing MB can cause serious ecological pollution and destruction. Furthermore, MB can result in adverse effects on human health, such as irreversible damage to the eyes, increased heart rate, vomiting, shock, an unhealthy pallor, jaundice, and tissue necrosis. Therefore, how to degrade MB in wastewater has been the direction explored by many researchers.

In this work, for the purpose of enhancing the photo quantum efficiency of ZnO, SnIn_4_S_8_@ZnO, Z-scheme heterojunction was synthesized using a convenient two-step hydrothermal approach. Furthermore, the photocatalytic activity of SnIn_4_S_8_@ZnO was assessed through the degradation of MB under the irradiation of ultraviolet (UV) light.

## 2. Materials and Methods

### 2.1. Synthesis of ZnO

ZnO in the form of nanosheets was prepared using a simple hydrothermal process. ZnCl_2_ (Bohuatong, Tianjin, China) aqueous solution (50 mL, 1 mol/L) and KOH (Tailande, Tianjin, China) aqueous solution (20 mL, 7.5 mol/L) were prepared, respectively. The above two solutions were mixed, agitated for 20 min, and then poured into a stainless-steel autoclave with Teflon lining whose volume was 100 mL. Afterward, the sealed autoclave was heated in the electric oven at 180 °C for 10 h and then naturally cooled to an ambient temperature. Finally, the product was separated through centrifugation, washed several times using deionized water and anhydrous alcohol (Bohuatong, Tianjin, China), dried at 60 °C for 10 h, and then ground for the formation of ZnO powders.

### 2.2. Synthesis of SnIn_4_S_8_@ZnO Heterojunction

**Figure 1:**
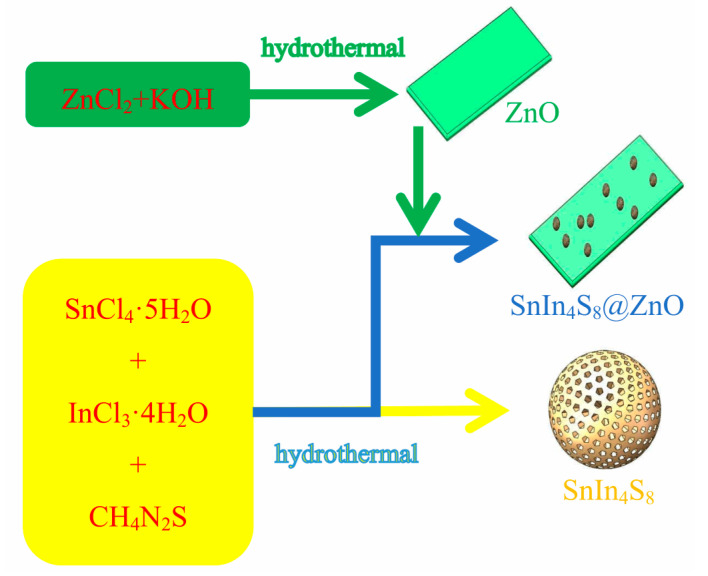
Synthesis chart of SnIn_4_S_8_, ZnO and SnIn_4_S_8_@ZnO.

The typical procedure for SnIn_4_S_8_@ZnO heterojunction synthesis was as follows. First, 70 mL of the anhydrous ethanol solution with a certain quantity of SnCl_4_·5H_2_O (Shanpu, Shanghai, China) was prepared. Second, a certain quantity of InCl_3_·4H_2_O (Macklin, Shanghai, China) was dissolved in the solution, which was stirred for 10 min. Third, 2.0345 g of the as-prepared ZnO was mixed into the solution, which was agitated for 20 min. Fourth, a certain quantity of CH_4_N_2_S (Beilian, Tianjin, China) was mixed into the solution which was agitated for 20 min. Fifth, the solution was poured into a stainless steel autoclave with a Teflon lining whose volume was 100 mL; the sealed autoclave was heated in an electric oven at 160 °C for 10 h and then naturally cooled to an ambient temperature. Finally, the products were washed using deionized water, dehydrated under a vacuum at 70 °C for 10 h, and then ground for further use. The obtained samples were recorded as ZSX (X = 200, 400, 600, 800 and 1000), where X represents the molar ratio of ZnO to SnIn_4_S_8_. In addition, SnIn_4_S_8_ was prepared through the same approach mentioned above, except for the addition of as-synthesized ZnO. The flow chart for the synthesis of SnIn_4_S_8_, ZnO and SnIn_4_S_8_@ZnO samples is exhibited in Figure 1.

### 2.3. Characterizations

The phase compositions of as-synthesized samples were analyzed via an X-ray diffractometer (XRD, Rigaku Smartlab3kw, Tokyo, Japan). The morphologies of the samples were described using a field emission scanning electron microscope (FESEM, Zeiss Sigma 300, Jena, Germany) and a transmission electron microscope (TEM, FEI Titan G2 60-300, Hillsboro, OR, USA). The distribution of elements was analyzed through an energy-dispersive X-ray (EDX) detector (Oxford X-Max, Oxford, UK), which was merged into the FESEM. The specific surface area and pore size distribution were obtained from nitrogen adsorption/desorption data recorded through an instrument (Micromeritics 3Flex, Norcross, GA, USA). A UV-visible spectrophotometer (Shimadzu UV-2550, Kyoto, Japan) was used to characterize the absorption spectra of specimens. A fluorescence spectrophotometer (Lengguang Tech. F97XP, Shanghai, China) was used to test the photoluminescence (PL) spectra of specimens. The total organic carbon (TOC) measurement was performed using a TOC analyzer (Shimadzu TOC-L, Kyoto, Japan).

### 2.4. Photocatalytic Degradation of MB

The photocatalytic efficiencies of specimens were evaluated by choosing MB as the model pollutant. In total, 15 mg of catalyst powders were added to the MB solution (100 mL, 10 mg/L). The quantities of SnIn_4_S_8_ in 15 mg of ZS200, ZS400, ZS600, ZS800 and ZS1000 were 0.72 mg, 0.36 mg, 0.25 mg, 0.18 mg and 0.15 mg, respectively. The mixture was treated for 10 min through ultrasonic vibration and then agitated for 30 min in the dark to reach an adsorption/desorption equilibrium. After that, the constantly stirred mixture was illuminated using an ultraviolet lamp whose power and irradiation peak wavelength were 36 W and 365 nm, respectively. In total, 5 mL of the mixture was extracted at an interval of 5 min, and then the photocatalyst was removed through centrifugation. Finally, the UV-Vis spectrophotometer (Aoyi Inst. UV-1800PC, Shanghai, China) was used to determine the absorbance of the solution at 664 nm.

## 3. Results and Discussion

### 3.1. XRD Analysis

**Figure 2:**
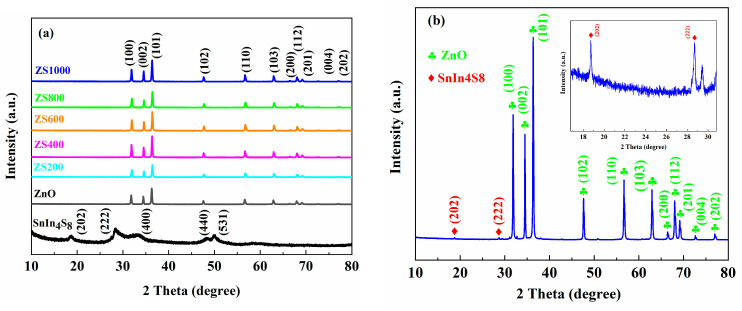
XRD spectra of (**a**) ZnO, SnIn_4_S_8_ and ZSX (scanning rate of 10°/min) and (**b**) ZS1000 (scanning rate of 4°/min).

XRD spectra of ZnO, SnIn_4_S_8_ and ZSX with a scanning speed of 10°/min are displayed in Figure 2a. Among them, the characteristic peaks of ZnO correspond to (100), (002), (101), (102), (110), (103), (200), (112), (201), (004) and (202) crystalline facets of the hexagonal wurtzite phase of ZnO (JCPDS card No. 36-1451) [31]. The characteristic peaks of SnIn_4_S_8_ at 2θ = 18.7°, 28.6°, 33.2°, 48.3° and 50.1°, which are consistent with the (202), (222), (400), (440) and (531) crystal faces of cubic phase of SnIn_4_S_8_ (JCPDS card No. 42-1305) can be observed separately [32]. Furthermore, no characteristic peaks of any foreign substance could be found in the XRD spectra, suggesting the excellent purity of the as-prepared SnIn_4_S_8_. In addition, the characteristic peaks of ZSX samples could be ascribed to the diffraction planes of ZnO (JCPDS card No. 36-1451). However, no diffraction peaks corresponding to SnIn_4_S_8_ were found in the XRD patterns of ZSX samples, which could be attributed to the low level of SnIn_4_S_8_ in ZSX specimens and the relatively fast scanning rate of XRD. In order to verify the existence of SnIn_4_S_8_ in ZSX samples, as-prepared ZS1000 was tested through XRD with a scanning rate of 4°/min, and the pattern (Figure 2b) clearly displays that the diffraction peaks at 18.7° and 28.6° can be ascribed to the (202) and (222) plane of SnIn_4_S_8_ (JCPDS card No. 42-1305).

### 3.2. Morphological Analysis

**Figure 3:**
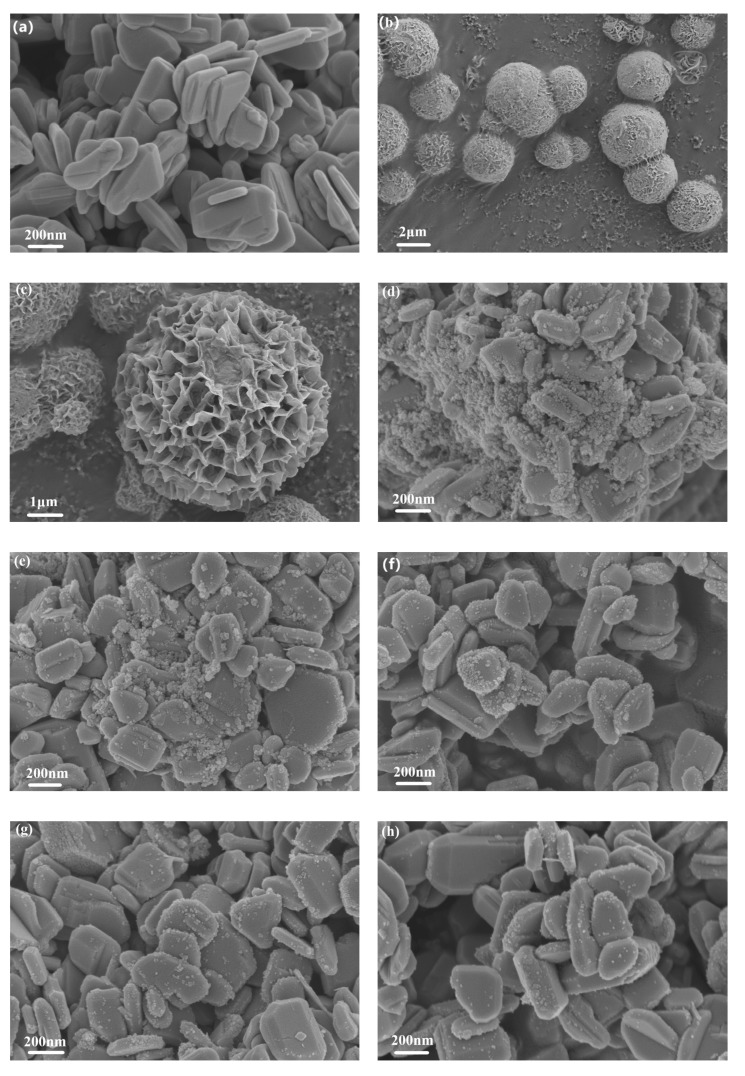
Microstructures of (**a**) Pure ZnO nanosheets, (**b**,**c**) SnIn_4_S_8_ microspheres, (**d**) ZS200, (**e**) ZS400, (**f**) ZS600, (**g**) ZS800 and (**h**) ZS1000 heterostructures.

**Figure 4:**
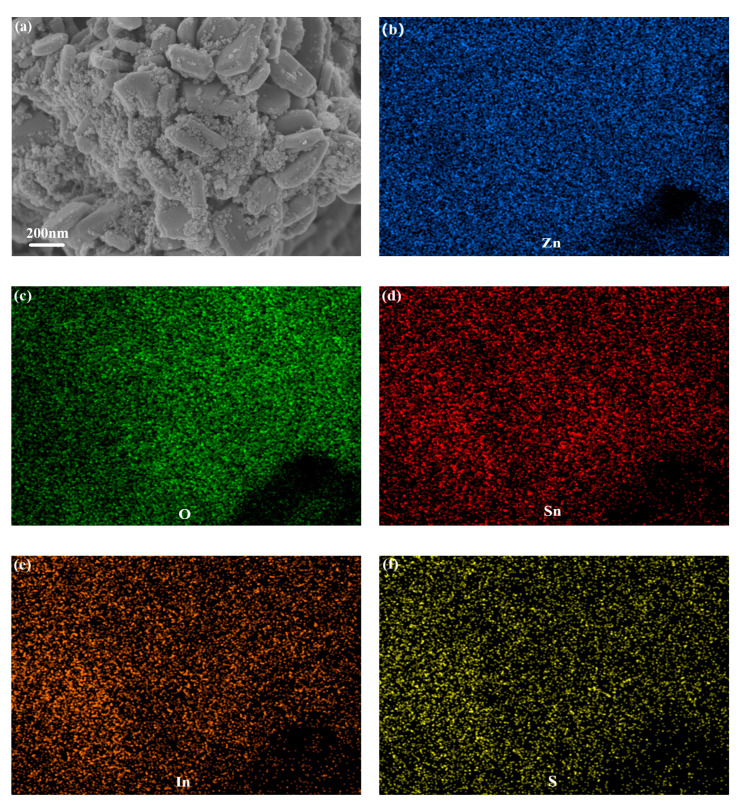
(**a**) Microstructure of ZS200 and (**b**–**f**) EDX elemental mapping images in the zone of (**a**).

The FSEM photographs of ZnO, SnIn_4_S_8_ and ZSX samples are presented in Figure 3. It can be seen that ZnO showed a flaky morphology with a smooth surface (Figure 3a). As-prepared SnIn_4_S_8_ showed the morphology of 3D hierarchical reticular microspheres with diameters at 2–4 μm (Figure 3b,c). From the magnified FSEM picture, it can be observed that the microspheres were composed of many two-dimensional nanoflakes with a thickness of 20 nm. Furthermore, the microspheres possessed a porous appearance because of the interlacing of nanoflakes. The SEM images of ZSX samples show that many SnIn_4_S_8_ nanoparticles were unevenly distributed on the surface of the ZnO Substrate (Figure 3d–h). Furthermore, the number of nanoparticles gradually decreased with the increase in the molar ratio of ZnO to SnIn_4_S_8_. Moreover, the EDX elemental mapping analysis of ZS200 demonstrated that the elements Zn, O, Sn, In and S existed in ZSX samples (Figure 4), which could further suggest that the large flake-like substrates were ZnO and the small nanoparticles were SnIn_4_S_8_.

**Figure 5:**
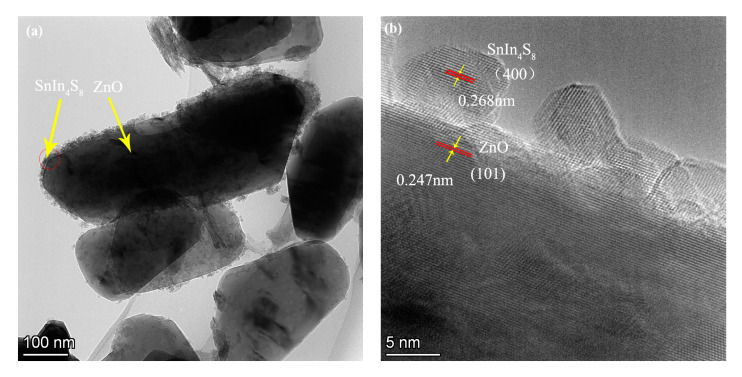
(**a**) TEM and (**b**) HRTEM photos of ZS800.

The TEM photo of ZS800 obviously indicates that the SnIn_4_S_8_ nanoparticles densely covered the surface of nanoflake-like ZnO to obtain the SnIn_4_S_8_@ZnO heterojunction (Figure 5a). Two kinds of lattice fringes are clearly displayed in the HRTEM image of ZS800 and as shown in Figure 5b, the observed interplanar spacing of 0.247 nm corresponded to the ZnO (101) plane (JCPDS card No. 36-1451), and the characteristic interplanar spacing of 0.268 nm belonged to the SnIn_4_S_8_ (400) plane (JCPDS card No. 42-1305) [31,33]. Furthermore, it can be observed that the contact interface between ZnO and SnIn_4_S_8_ was very tight. The results mentioned above demonstrate that the SnIn_4_S_8_@ZnO heterojunction was successfully constructed. The tight contact interface between SnIn_4_S_8_ and ZnO is beneficial for the migration of photogenerated charge carriers and the stability of the structure. As a consequence, the SnIn_4_S_8_@ZnO heterojunction can exhibit enhanced photocatalytic activity.

### 3.3. BET Surface Area Analysis

**Figure 6:**
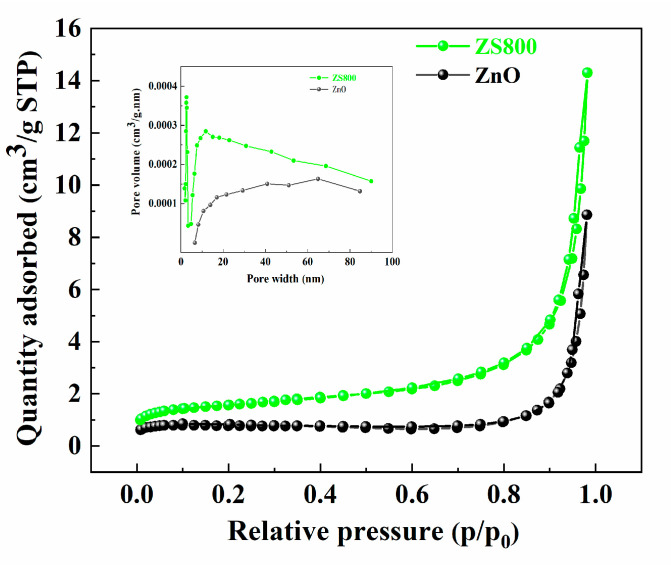
N_2_ adsorption/desorption isotherms and the corresponding pore size distribution curves (inset) of ZnO and ZS800.

The nitrogen adsorption/desorption and pore size distribution curves of ZnO and ZS800 are displayed in Figure 6. The isotherms of two catalysts can be identified as type IV, indicating the existence of mesopores. Furthermore, it was found that the nitrogen adsorption capacity of ZS800 was clearly stronger than that of ZnO because the specific surface area of ZS800 was larger than that of ZnO. The specific surface area, pore volumes, and average pore sizes of the two samples are summarized in Table 1. Compared to ZnO, ZSX800 possessed a larger specific surface area and pore volume, resulting in the fact that ZSX800 had more active sites and a stronger adsorption capacity for MB, which is beneficial for the improvement of photocatalytic activity.

### 3.4. UV-Vis Absorption Spectra Analysis

**Figure 7:**
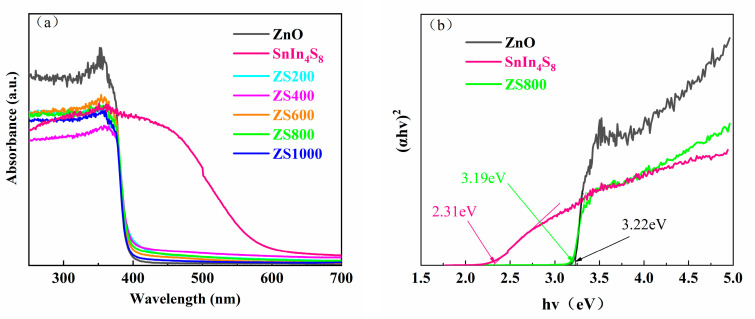
(**a**) UV-vis absorption spectra of ZnO, SnIn_4_S_8_ and ZSX. (**b**) Curves of (αhv)^2^ versus the hv of ZnO, SnIn_4_S_8_ and ZS800.

The light-harvesting ability can be regarded as a crucial factor for the photocatalytic activity of semiconductors. It was found from the UV-vis absorption spectra (Figure 7a) that all the samples exhibited strong optical absorption in the region of UV. Moreover, the ZSX samples exhibited absorption cutoff wavelengths with slight redshift, and improvements in absorption capability in the Vis range were observed compared to pure ZnO. In addition, it was found that SnIn_4_S_8_ exhibited an excellent absorption capability in the Vis range. The band gap (E_g_) of crystalline semiconductors can be obtained using the Kubelka:

(1) $$\alpha hv={A(hv-E_{g})}^{n/2}$$

where α is the absorbance, hv is the photon energy, and A is a constant [34]. The selection of n value (1 for direct transition, while 4 for indirect transition) is based on the transition property in the semiconductor [35]. The band gap energy of as-synthesized specimens was determined by selecting n as one because both SnIn_4_S_8_ and ZnO are direct transition semiconductors. Figure 7b presents the (αhν)^2^ curves with a hv of ZnO, SnIn_4_S_8_ and ZS800, and the band gaps can be calculated by lengthening the linear portion to the x-axis, which indicates that the band gaps of ZnO, SnIn_4_S_8_ and ZS800 were about 3.22 eV, 2.31 eV and 3.19 eV, respectively. The narrowing of bandgap energy suggested that the light-harvesting capability of ZnO was improved by hybridization with SnIn_4_S_8_.

### 3.5. PL Spectra Analysis

**Figure 8:**
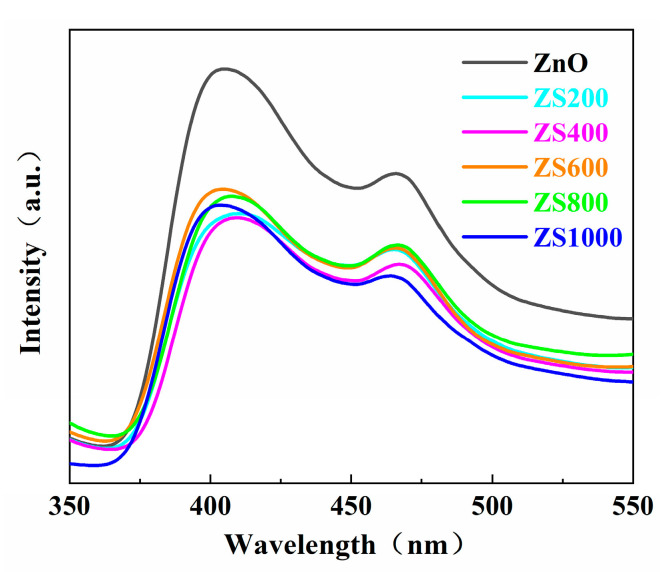
PL spectra of ZnO and ZSX.

The recombination rate of photoinduced charge carriers is reflected through the peak intensity of the PL spectrum. Normally, a weaker peak intensity reflects a higher separation efficiency in the photogenerated charge carriers, which results in better photocatalytic performance [36,37]. The PL spectra of the as-synthesized ZnO and ZSX specimens are displayed in Figure 8, which reveals that all specimens had similar PL spectra with two distinct luminescence peaks at approximately 400 and 460 nm. The peak at 400 nm was the emission near the edge of the band, which originated from the recombination of free excitons through the collision between the excitons, and the peak at about 460 nm could be the result of the inherent defects of ZnO [38,39]. Furthermore, compared with ZnO, the intensity of the fluorescence spectra of ZSX samples was significantly reduced. It indicates that the SnIn_4_S_8_@ZnO heterojunction had a lower recombination rate of photogenerated charge carriers, which is beneficial for the improvement of the photocatalytic performance of the catalyst [40].

### 3.6. Photocatalytic Activities of the Specimens

**Figure 9:**
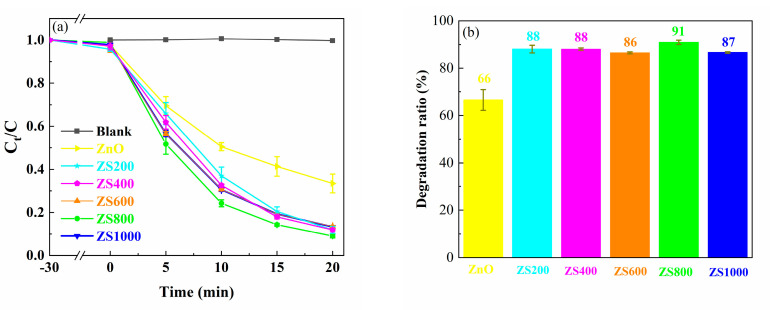

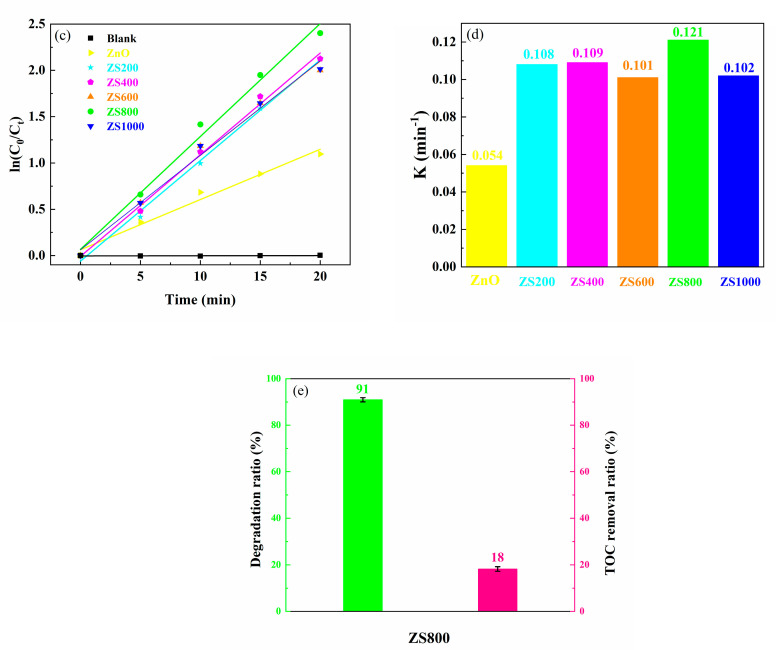
(**a**) Degradation curves of MB solution, (**b**) Ultimate degradation ratios of MB after 20 min, (**c**) Kinetic curves of MB degradation, (**d**) Calculated k values of MB degradation and (**e**) Ultimate degradation and TOC removal ratios of MB after 20 min.

The MB photodegradation curves with ZnO, ZSX samples and no photocatalyst are shown in Figure 9a, and the ultimate degradation rates of MB after 20 min are exhibited in Figure 9b. The concentration of MB was nearly unchanged during the illumination process without a photocatalyst, meaning that MB could not be removed through photolysis. The changes in MB concentrations were very small when MB was adsorbed for 30 min using all samples in the dark, which indicated that the adsorption effects of the ZnO and ZSX samples on MB were negligible. After 20 min of UV illumination, the degradation ratios of MB degraded by ZnO, ZS200, ZS400, ZS600, ZS800 and ZS1000 were 66%, 88%, 88%, 86%, 91% and 87%, respectively. Compared with the ZnO, the ZSX samples exhibited clearly improved photocatalytic activities. Furthermore, ZS800 had the highest photocatalytic efficiency for MB degradation.

Generally speaking, the photodegradation of MB complies with the pseudo first-order kinetic equation:

(2) $$\ln\left(\frac{C_{0}}{C_{t}}\right)=kt$$

where k, t, C_0_, and C_t_ are the apparent rate constant, and the photocatalytic reaction time, the MB concentrations at the moment of t = 0 and at a certain time, respectively [41]. The kinetic curves of MB photodegradation in the presence of ZnO, ZSX samples and no photocatalyst were fitted by plotting ln(C_0_/C_t_) versus time (Figure 9c), and a good linear relationship was observed. The gradient of the kinetic curve denotes the k value, which corresponds to the degradation efficiency. The k values of MB degradation over ZnO, ZS200, ZS400, ZS600, ZS800 and ZS1000 were 0.054, 0.108, 0.109, 0.101, 0.121 and 0.102 min^−1^, separately (Figure 9d). It was found that the k value of ZSX samples for MB degradation was obviously higher than that of ZnO. Furthermore, ZS800 had the highest k value, which was approximately 2.2 times that of ZnO. These results indicate that the photocatalytic activity of ZnO can be significantly boosted through hybridization with SnIn_4_S_8_ for the formation of the SnIn_4_S_8_@ZnO heterojunction, which is an efficient photocatalyst.

In addition, in order to estimate the mineralization ability of the photocatalyst, TOC removal efficiency after 20 min of MB degradation using ZS800 was measured. As displayed in Figure 9e, TOC removal efficiency after 20 min of MB degradation using ZS800 was 18%. This result indicates that the photocatalytic degradation of MB using ZSX samples can be accompanied by partial mineralization.

**Figure 10:**
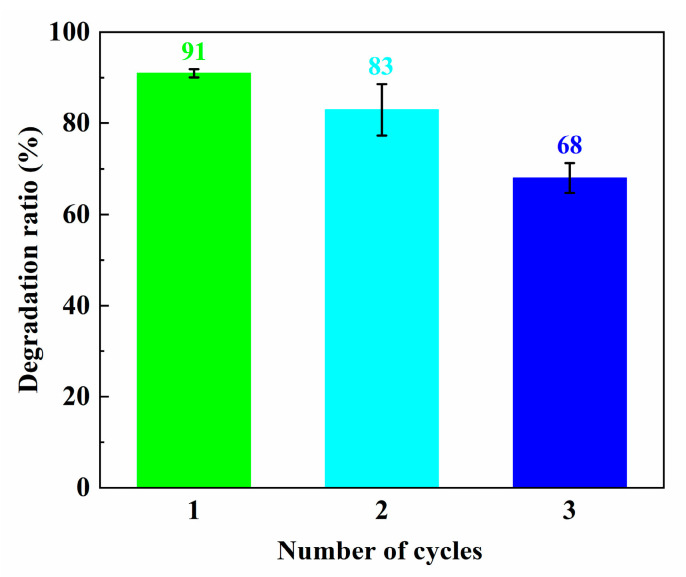
Final degradation ratios of MB over ZS800 for 20 min in three cycles.

**Figure 11:**
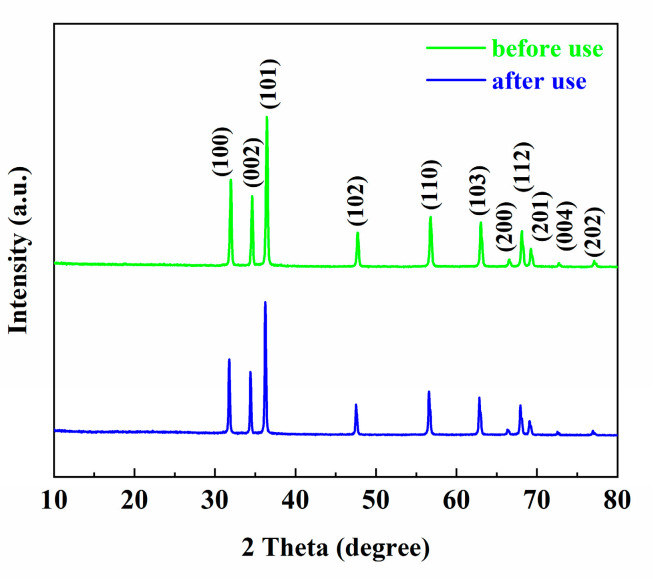
XRD spectra of ZS800 before and after three cycles.

**Figure 12:**
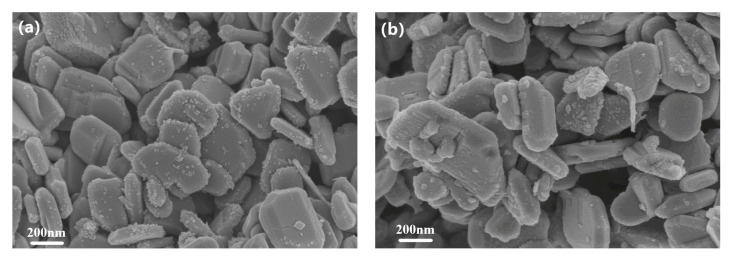
Microstructures of ZS800 (**a**) Before and (**b**) After three cycles.

In order to check the stability and reusability of catalysts, the ZS800 was used to degrade MB for three cycles. The final degradation ratios of MB over ZS800 in 20 min for three cycles are shown in Figure 10. The degradation ability of ZS800 for MB slightly decreased after three cycles, with about a 23% decrease in MB degradation from 91% to 68%, indicating that as-prepared catalysts were relatively stable for practical application. The XRD and SEM results before and after three cycles are also provided in Figure 11 and Figure 12, from which it can be found that ZS800 did not change obviously. The slight decrease in photocatalytic activity might be due to a decrease in surface-active sites.

**Figure 13:**
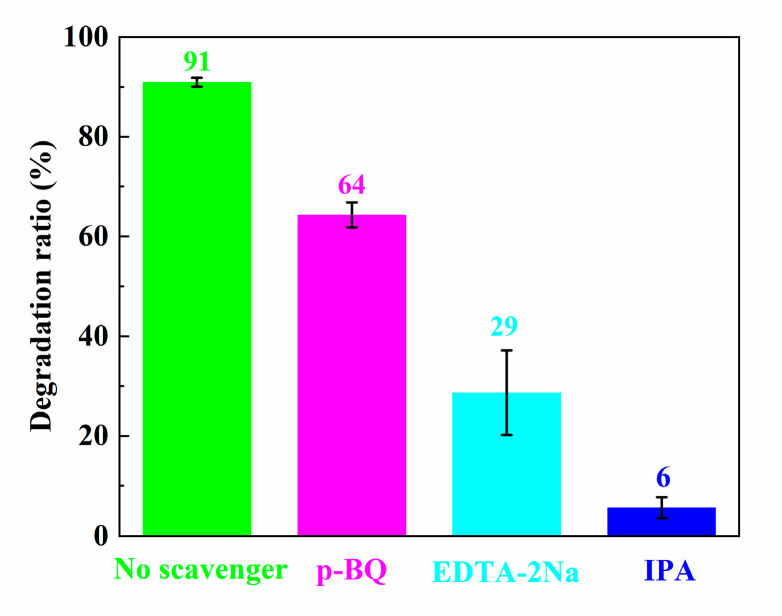
Final degradation ratios of MB over ZS800 for 20 min with different scavengers.

To probe reactive species for the degradation of the MB solution during the photocatalytic process, the trapping experiment of ZS800 was carried out, where isopropyl alcohol (IPA, Tianli, Tianjin, China), p-benzoquinone (p-BQ, Macklin, Shanghai, China) and EDTA-2Na (Tianli, Tianjin, China) were utilized to trap ·OH, ·O_2_^−^ and h^+^, respectively. As shown in Figure 13, the degradation ratios of MB were apparently inhibited to 6% and 29% when IPA and EDTA-2Na were added into the solution, while the degradation ratio of MB was 64% with a relatively insignificant reduction after adding p-BQ. These results indicate that ·OH and h^+^ were the main active species for this photodegradation process, while ·O_2_^−^ was not the dominant reactive species.

### 3.7. Photocatalytic Mechanism Analysis

**Figure 14:**
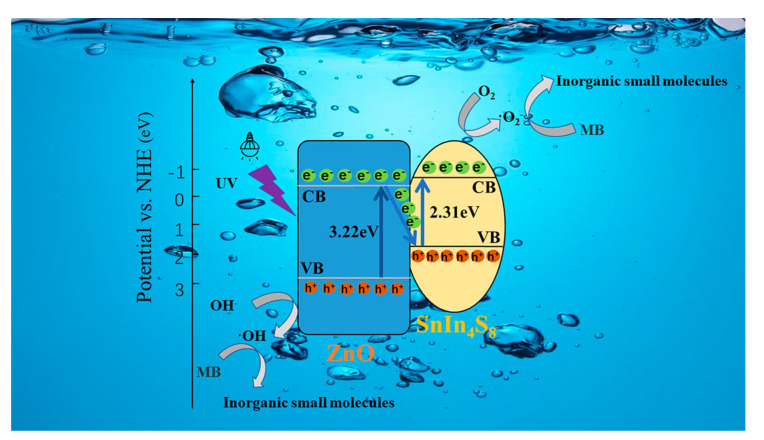
Sketch map of the carriers of migration and photocatalysis.

According to the results of this work and several previous investigations, a Z-scheme mechanism can be proposed for the efficient degradation of MB using SnIn_4_S_8_@ZnO heterojunction, as shown in Figure 14. Based on the above results of UV-Vis absorption spectra analysis, the band gaps (E_g_) of ZnO and SnIn_4_S_8_ were 3.22 eV and 2.31 eV, respectively. In addition, the reported conduction band potentials (E_CB_) of ZnO and SnIn_4_S_8_ were −0.31 eV and −0.52 eV, respectively (vs. NHE) [42,43]. The valence band potentials (E_VB_) of ZnO and SnIn_4_S_8_ can be calculated according to Equation (3) [44,45]:

(3) $$E_{\mathrm{VB}}=E_{\mathrm{CB}}+E_{g}$$

The calculated E_VB_ of ZnO and SnIn_4_S_8_ were 2.91 eV and 1.79 eV, respectively (vs. NHE). Therefore, it can be concluded that the conduction band (CB) position of SnIn_4_S_8_ was higher than that of ZnO, while the valence band (VB) position of ZnO was lower than that of SnIn_4_S_8_. Under the irradiation of UV light, photoinduced electrons can transfer from the VB of ZnO and SnIn_4_S_8_ to the CB, respectively, keeping the photogenerated holes in the VB of them. Then, the photoinduced electrons in the CB of the ZnO can migrate to the VB of SnIn_4_S_8_ owing to an appropriate potential difference. Eventually, a Z-shaped migration path of photogenerated electrons is formed in the SnIn_4_S_8_@ZnO heterojunction. This mechanism results in the fact that the photoinduced electrons collect in CB of SnIn_4_S_8_ while the photoinduced holes gather in VB of ZnO. These photoexcited electrons and holes are efficiently separated on the opposite side of the SnIn_4_S_8_@ZnO heterojunction. Therefore, the life span of photo-induced carriers can be greatly improved, leading to a remarkable improvement in photocatalytic activity. The photoinduced holes in VB of ZnO are vital oxidants; they can quickly oxidize hydroxyl anions (OH^−^) to hydroxyl radicals (·OH). The photogenerated electrons in CB of SnIn_4_S_8_ are robust reductants; they can rapidly reduce dissolved oxygen (O_2_) into superoxide radicals (·O_2_^−^). Finally, the MB molecules can be degraded into inorganic small molecules by these highly active ·OH, h^+^ and ·O_2_^−^ species. Overall, the main reactions of this mechanism can be expressed as follows:

(1) $ZnO/{SnIn}_{4}S_{8}+hv\to ZnO(h^{+}\left(VB\right)+e^{-}\left(CB\right))/{SnIn}_{4}S_{8}(h^{+}\left(VB\right)+e^{-}\left(CB\right))$;
(2) $ZnO(h^{+}\left(VB\right)+e^{-}\left(CB\right))/{SnIn}_{4}S_{8}(h^{+}\left(VB\right)+e^{-}\left(CB\right))\to ZnO(h^{+}\left(VB\right))+{SnIn}_{4}S_{8}(e^{-}\left(CB\right))$;
(3) $ZnO(h^{+}\left(VB\right))+{OH}^{-}\;\to \;\cdot OH$;
(4) ${SnIn}_{4}S_{8}(e^{-}\left(CB\right))+O_{2}\;\to \;\cdot O_{2}^{-}$;
(5) $\cdot OH,ZnO\left(h^{+}\left(VB\right)\right),\cdot O_{2}^{-}+MB\;\to \;inorganic\;small\;molecules$.

## 4. Conclusions

In this work, the SnIn_4_S_8_@ZnO Z-scheme heterojunction photocatalyst was successfully acquired through a convenient two-step hydrothermal approach. The as-synthesized SnIn_4_S_8_@ZnO exhibited significantly enhanced photocatalysis activity under UV light irradiation. The degradation ability of the SnIn_4_S_8_@ZnO structure toward MB was significantly stronger than that of pure ZnO. The remarkable performance of MB photodegradation can be ascribed to the efficient spatial separation of photoinduced electrons and holes through a Z-scheme heterojunction with an intimate contact interface. To conclude, the results in this paper bring a novel insight into constructing excellent ZnO-based photocatalytic systems for wastewater purification.

## Figures and Tables

**Table 1:** Surface and porosity properties of ZnO and ZS800.

Samples	Specific Surface Area (m^2^/g)	Pore Volume (cm^3^/g)	Average Pore Size (nm)
ZnO	3.2329	0.0137	17.0021
ZS800	5.6337	0.0221	15.7473

## Data Availability

The data presented in this study are available on request from the corresponding author.
